# Loss of the TNFα function inhibits Wnt/β-catenin signaling, exacerbates obesity development in adolescent spontaneous obese mice

**DOI:** 10.1007/s11010-014-1987-5

**Published:** 2014-02-13

**Authors:** Maolei Gong, Chuanguo Liu, Liang Zhang, Hongbin Zhang, Jie Pan

**Affiliations:** 1 Shandong Provincial Key Laboratory of Animal Resistant Biology, College of Life Sciences, Shandong Normal University, #88 East Wenhua Ave., Jinan, 250014 People’s Republic of China; 2 Department of Biology, University of Copenhagen, 2100 Copenhagen, Denmark

**Keywords:** TNFα, Wnt/β-catenin signaling, Leptin receptor, Obesity, Adipocyte

## Abstract

Tumor necrosis factor alpha (TNFα) is an adipokine involved in the regulation of cell differentiation and lipid metabolism, but its specific role has not been clearly understood. We validated a hypothesis that loss of TNFα function would inhibit Wnt/β-catenin signaling and accelerate adipogenesis in adolescent genetic obese mice. Epididymal white adipose tissues (eWAT) from TNFα deficient (TNFα^−/−^), leptin receptor deficient (*db/db)* and double gene mutant (*db/db*/TNFα^−/−^, DT) male mice were used for comparative analysis of key molecules in Wnt/β-catenin signaling and adipogenic markers by qRT-PCR and western blot techniques. Compared with TNFα^−/−^ and WT mice of 28 days old, an obese trait was observed in both *db/db* and DT mice, while the latter showed more significant body weight gain and eWAT hypertrophy. The mRNA level of key molecules in Wnt/β-catenin pathway was reduced in both obese groups, while the DT group was the lowest. Expression of adipocyte-specific genes was up-regulated during obese development in the two obese groups, while the DT group revealed more correlation than that of *db/db* group. At the protein level, a down regulation of Wnt10b and β-catenin in obese eWAT showed similar tendency with that of mRNA level. Compared with the lean groups, the levels of adiponectin and PPARγ2 for the obese groups were down-regulated at 21-day-old age, while they were elevated at older age. Our results suggested that deficiency in TNFα inhibited Wnt/β-catenin signaling of the obese eWAT and up-regulated expression of adipokines, and accelerated adipogenesis in genetic obese mice on a chow diet.

**Electronic supplementary material:**

The online version of this article (doi:10.1007/s11010-014-1987-5) contains supplementary material, which is available to authorized users.

## Introduction

Recent studies have indicated that obesity in both human and animal is actually a state of chronic low--grade inflammation, which is characterized by the increase of local and circulating proinflammatory adipokines secreted mainly by enlarged visceral white adipocytes, including tumor necrosis factor alpha (TNFα), interleukins (ILs), leptin, and adiponectin [1–3]. Hereinto, as a part of the inflammatory cascade, TNFα is a chief and pleiotropic cytokine involved in the regulation of cell proliferation, differentiation, lipid metabolism, and obesity [1, 4–12].

It was reported that TNFα not only impaired adipocyte differentiation through stimulating leptin production [7], but also induced lipolysis [8], impaired lipogenesis, and lipid accumulation [3, 9–11] and reduced adiponectin production [12]. Besides, TNFα also promoted an inflammatory state and local secretion of cytokines in white adipose tissue, such as IL-1 and IL-6 [1, 3, 12]. In numerous experiments, including our pervious work, however, TNFα deficient (TNFα^−/−^) mice showed normal body weight and visceral and subcutaneous adipose mass with down regulation of inflammatory cytokines after being fed with a chow diet for 6 months [4, 6], although the body weight was elevated after TNFα^−/−^ mice were fed with a high carbohydrate diet for several months [6]. Therefore, it was considered that activation of the TNFα system in obesity was a key action to inhibit ongoing weight gain [13]. We have previously demonstrated that TNFα^−/−^ mice on apolipoprotein E mutant (apoE^−/−^) background reduced the formation of lipid accumulating foam cells in atherosclerotic plaques, and influenced expression of proinflammatory markers in aorta [4]. However, weight gain of white adipose tissue was not observed in mice with TNFα deficiency alone or TNFα deficiency on apoE^−/−^ background [6]. These results indicated that TNFα deficiency might affect obese subjects rather than lean individuals in terms of genetic background or diet induced obesity, since the results advanced differentiation and maturation of the adipocytes.

Wnt/β-catenin signaling is an endogenous inhibitory force, as a predominant regulator of adipose biology [14–17]. Recently, it has been reported that adipose TNFα plays an important role in the regulation of normal adipocyte differentiation, and that lipid accumulation is inhibited by a maintained Wnt signaling in preadipocytes [14]. Obesity associated inflammation leads to highly dysregulated white adipose tissues with an altered pattern of secreted adipokines and increased lipolysis [18]. However, results and conclusions so far from cohort studies have not been consistent with each other. Then, the following concerns are raised: (i) Does elevated TNFα in obese subjects play as a secondary response to obesity? (ii) Does loss of TNFα function influence adipogenesis in genetic obesity background during development of obesity on a chow diet?

The aim of this study mainly focused on the effect of TNFα on the development of adiposity in genetic obesity-prone young mice. Besides, whether TNFα deficiency influenced body weight gain of the obese mice on a chow diet indeed through inhibiting Wnt/β-catenin signaling in visceral adipose tissue and promoting adipogenesis was also discussed and analyzed.

## Materials and methods

### Animals

Leptin receptor mutant heterozygous (*Lepr* ^*db/*+^) mice and TNFα^−/−^ mice (both in C57BL6/J genetic background) were purchased from the Jackson Laboratory (Bar Harbor, ME, USA). Homozygous *Lepr* ^*db/db*^ (*db/db*) mice were obtained by interbred within *Lepr* ^*db/*+^ mice. *Lepr* ^*db/*+^ and TNFα^−/−^ mice were crossbred to generate double gene mutant (*db/db*/TNFα^−/−^, DT) mice. Genotypes of all mice were confirmed using polymerase chain reaction (PCR) technique. As previously observed, both obese *db/db* and DT mice revealed significant obese trait since 28 days of age. Therefore, to investigate relationship between TNFα deficiency and adipose development at young age, 21- and 42-day-old mice were used in this study. Wide-type (WT) C57BL6/J mice were used for normal control. Four groups of male mice were fed with a standard chow diet (4 % fat and 0.075 % cholesterol) and water ad libitum from 21 to 42 days of age. The mice were maintained at 22 ± 1 °C with 12-h light and 12-h dark cycles in our specific pathogen-free animal facility, and their body weight was measured every 3 days.

Epididymal visceral white adipose tissues (eWAT) were collected for analyzing morphological characteristics and expression pattern of key molecules in Wnt/β-catenin signaling and adipogenic markers, and multiple techniques were used in order to obtain sufficient information.

### Measurement of plasma lipids, glucose, and insulin

After 12-h fasting before sacrifice at various ages, four groups (*n* = 10 in each group per age) of peripheral blood were obtained. Plasma concentrations of total cholesterol (TC), triglycerides (TG), low-density lipoprotein cholesterol (LDL-C), high-density lipoprotein cholesterol (HDL-C), and glucose (GLU) were measured using COD-PAP and GPO-PAP methods with automatic analyzer BAYER ADVIA-2400 (Germany), as described in Ref. [19]. The plasma insulin concentration was determined using ultrasensitive ELISA kit (Kongcheng, Shanghai, China).

### Histological analysis

Epididymal visceral white adipose tissues from four groups at various ages (*n* = 10 in each group per age) were fixed in 10 % neutral buffered formalin and cryostat sections (6–8 μm, 20 sections of each group for each time point, respectively) were stained with hematoxylin and eosin (H & E). Images were randomly captured on an Olympus IX-71 microscope with an Olympus DP71 color digital camera. MetaMorph software (version 6.1; Molecular Devices, Downingtown, PA, USA) was used to calculate cellular area adipocytes per field with integrated morphometry analysis.

### Gene expression analysis

Quantitative real-time reverse-transcribed PCR (qPCR) was performed as described in our earlier report [4]. Briefly, after mice (*n* = 12 in each group per time point) were sacrificed, total RNA of eWAT was extracted using Trizol^®^ reagent (Invitrogen, USA) and treated with DNase I (Promega, USA) at 37 °C for 30 min, and then 2 μg of each sample was reverse-transcribed using Quant RTase (Promega, USA) to cDNA. PCR primers (supplementary Table 1) for target genes, mainly included genes in Wnt/β-catenin signal pathway (Wnt10b and β-catenin) and early-, intermediate-, and post/later-stage of differential markers such as C/EBPα, C/EBPβ, adiponectin, lipoprotein lipase (LPL), PPARr2, fatty acid synthases (FAS), and acetyl-CoA carboxylase (ACC1). Beta-actin was as an internal standard to normalize the amount of cDNA in each sample. Results were analyzed using the Rotor-gene Real-Time Analysis Software 6.0.

### Western blot analysis

Total proteins of eWAT from the four groups (*n* = 12 in each group per time point) were extracted using a tissue protein extraction reagent (Kangchen Bioscience, China) as described earlier [19], and pooled to 3 sets for each group per time point. The samples were loaded on polyacrylamide gels and transferred to immobilon polyvinyldifluoride membranes (Hybond; Amersham Biosciences). After blocking in 5 % de-fat milk for 1 h at room temperature, the blots were probed for anti-Wnt10b, anti-β-catenin, anti-C/EBPβ, anti-PPARγ2, anti-IL-6 (R&D Systems, Abingdon, UK), and anti-adiponectin (Abcam, USA), respectively. The membranes were then incubated with horseradish peroxidase-conjugated secondary antibody. The blots were visualized by enhanced chemiluminescence reagents and exposited on Kodak X-Omat BT films (Eastman Kodak, USA). The expression of proteins was normalized by the expression of glyceraldehyde-3-phosphate dehydrogenase (GAPDH). Densitometry was performed using Image-Pro Plus 6.0 (Media Cybernetics, USA).

### Statistical analysis

All experiments were repeated for at least 5 times, and the data were all expressed as the form of mean ± SEM. The comparisons between groups were performed by one-way ANOVA with post hoc LSD *t* test, and a *P* value < 0.05 indicated a significant difference between the groups. A standard software package (SPSS for Windows 15.0) was used.

## Results

### Body weight and morphological characterization of eWAT

**Fig. 1:**
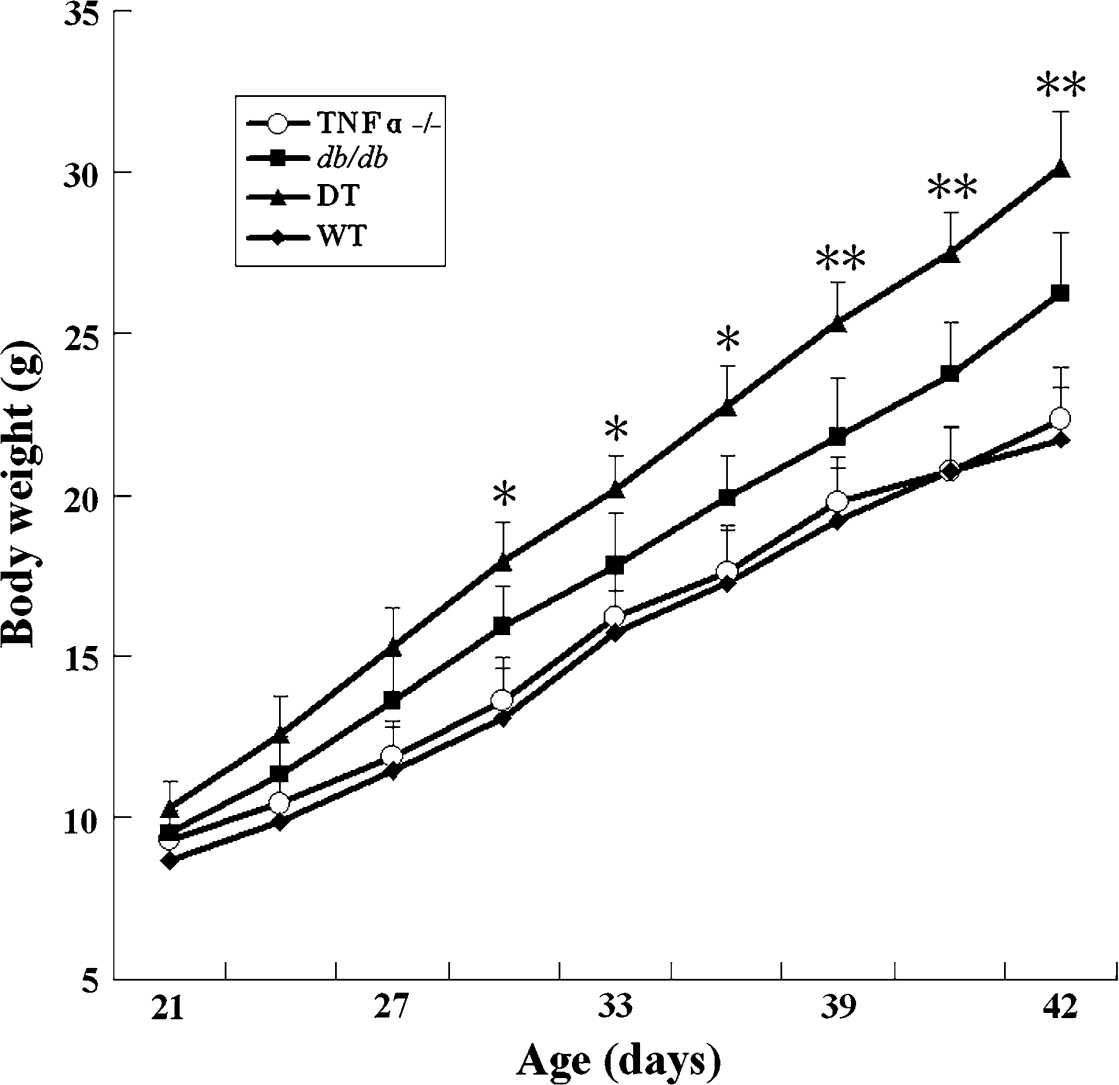
Body weight of the four genotypic mice. Body weight was measured for the four genotypic mice every 3 days from 21- to 42-day-old. Body weights were higher in DT mice than in *db/db* mice from 28-day-old, and there was a significant difference from 35-day-old (*P* < 0.05). Both DT and *db/db* mice were significantly higher than those of TNF-α^−/−^ and WT mice from 28- to 42-day-old (*P* < 0.05). Data were shown as the form of mean ± SEM. N ≥ 24 for each group. *P < 0.05, **P < 0.01

**Fig. 2:**
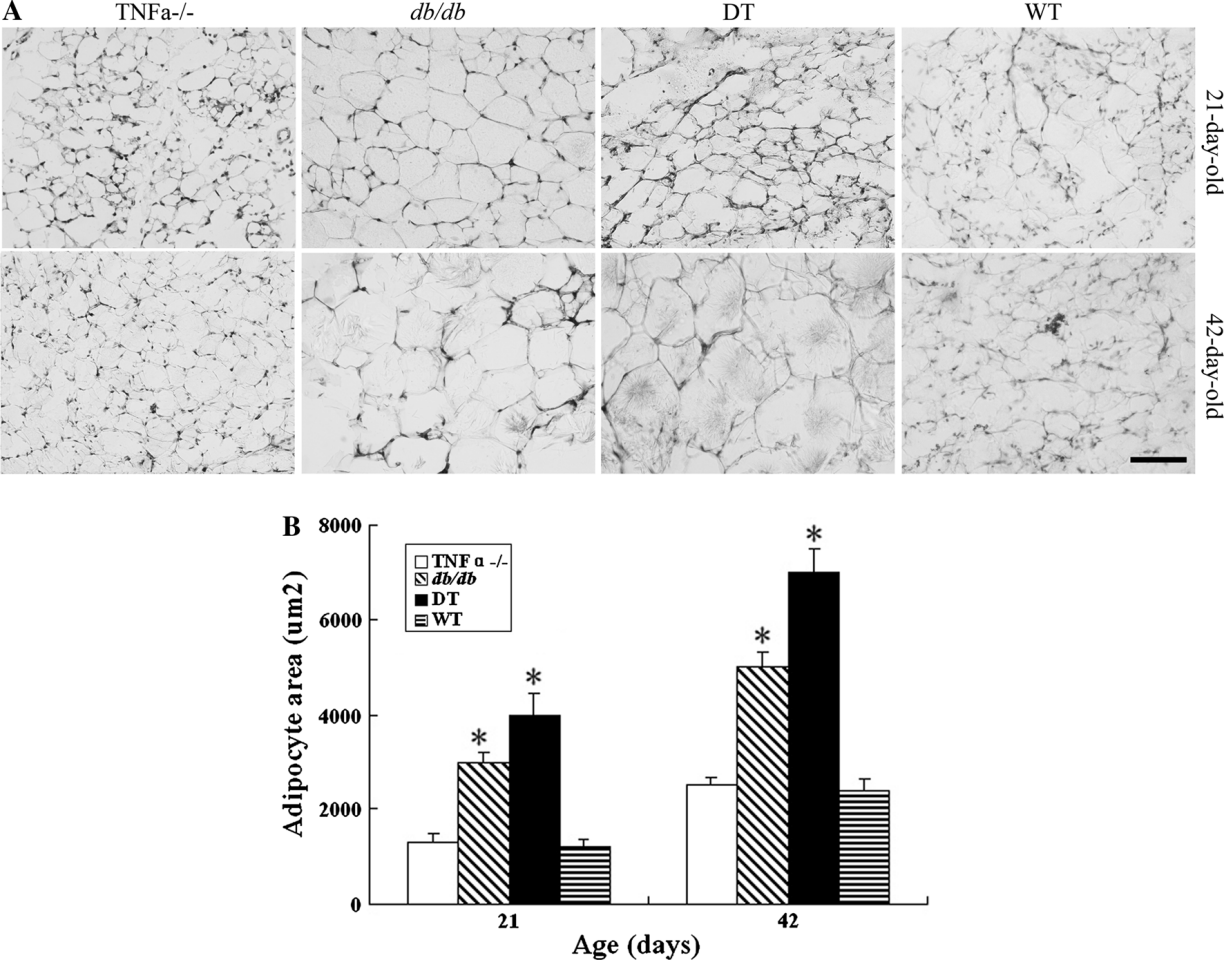
Morphology characterization of eWAT in *db/db*, DT, TNF-α^−/−^, and WT mice. Notably, adipocytes from obese (DT and *db/db*) mice were larger than those of lean (TNF-α^−/−^ and WT) mice at 21- and 4-day-old (**a**). Compared with *db/db* mice, the adipocytes from DT mice were more hypertrophic at age of 42 days and much more than those of lean mice (**b**). Twenty sections from each group at each time point were used for analysis

As shown in Fig. 1, body weight of obese (*db/db* and DT) mice is higher at 21-day-old, and significantly gains from 28-day-old compared with lean (TNFα^−/−^ and WT) mice. Notably, body weight of DT mice is significantly higher than that of *db/db* mice at 42-day-old (29 ± 0.6 g vs. 24 ± 0.5 g). Besides, the eWAT weight of DT mice is also higher than that of *db/db* mice, which is much more than that of lean mice at 42-day-old (Table 1). Moreover, adipocyte size of eWAT from obese mice is remarkably larger than that of lean mice at all time points (Fig. 2a), and average eWAT cell size of DT mice is significantly larger than that of *db/db* mice (Fig. 2b).

**Table 1:** eWAT weights of mice used in gene and protein expression analyses

	TNFα^−/−^	*db/db*	DT	WT
21-day-old				
eWAT (g)	0.10 ± 0.01^##^	0.41 ± 0.04	0.59 ± 0.03^#^	0.09 ± 0.01^##^
eWAT/BW (%)	1.1 ± 0.1^##^	4.3 ± 0.3	5.6 ± 0.2^#^	1.1 ± 0.1^##^
42-day-old				
eWAT (g)	0.22 ± 0.05^##^	2.38 ± 0.35	3.62 ± 0.31^#^	0.2 ± 0.02^##^
eWAT/BW (%)	1.2 ± 0.2^##^	9.8 ± 0.4	12.3 ± 0.3^#^	1.1 ± 0.09^##^

*n* = 12 mice per group

*eWAT* epididymal visceral white adipose tissues, *BW* body weight

^#, ##^Different letters indicate significant differences to *db/db* mice (*P* < 0.05 and *P* < 0.01, respectively)

By the way, in order to clarify that the body weight gain was mainly caused by white adipocyte hypertrophy, we also measured the weight of liver, and the result showed no significant difference between the two groups of obese mice, although there were significant weight gain and liver steatosis of both two obese groups compared with those of lean mice (data not shown).

### Plasma lipid parameters and glucose homeostasis

The plasma lipid parameters of each group are shown in Table 2. At age of 42 days, the levels of TC, TG, LDL-C, and HDL-C of DT mice are all higher than those of *db/db* mice (*P* < 0.05). Compared with WT control, TC levels of other three groups begin to increase from 42-day-old (*P* < 0.05). Moreover, TNFα^−/−^ and WT mice remain the euglycemic state all the time during the experimental period. There is no significant difference in plasma glucose among the four genotypes at age of 21 days, but the glucose levels of the obese mice are significantly higher than those of lean mice at 42-day-old (*P* < 0.01), and even higher in DT obese mice. Accordingly, *db/db* mice reveal higher level of plasma insulin at each point of age than DT mice do, and the insulin concentration of DT mice is lower than that of *db/db* mice, especially at 42-day-old (*P* < 0.01). The insulin level has no significant difference between TNFα^−/−^ and WT mice.

**Table 2:** Plasma lipids, glucose, and insulin concentration in four genotypic mice

Parameters (mM)	Age (days)	TNFα^−/−^	*db/db*	DT	WT
TC	21	2.68 ± 0.27^Δ^	2.15 ± 0.16	2.29 ± 0.37	2.22 ± 0.27
	42	3.15 ± 0.29*	3.38 ± 0.17*	3.33 ± 0.11**	2.36 ± 0.08
TG	21	1.05 ± 0.22^Δ^	0.61 ± 0.09	0.92 ± 0.18^Δ^	0.61 ± 0.12
	42	1.34 ± 0.13	1.05 ± 0.04	0.90 ± 0.08	1.37 ± 0.07
LDL-C	21	0.69 ± 0.15^Δ^	0.31 ± 0.12	0.33 ± 0.16	0.52 ± 0.08
	42	0.79 ± 0.14^Δ^	0.41 ± 0.10	0.56 ± 0.11	0.43 ± 0.07
HDL-C	21	1.81 ± 0.19	1.72 ± 0.11	1.62 ± 0.06	1.66 ± 0.32
	42	2.38 ± 0.25	2.71 ± 0.08	2.65 ± 0.05	2.00 ± 0.05
Glucose	21	4.01 ± 0.07	4.12 ± 0.05	3.95 ± 0.06	4.08 ± 0.07
	42	4.15 ± 0.1^Δ^	5.78 ± 0.15*	8.35 ± 0.12**^Δ^	4.13 ± 0.08
Ins (μU/ml)	21	65 ± 4.3^Δ^	95 ± 3.1*	80 ± 4.6**	65 ± 3.8^Δ^
	42	29 ± 3.7^Δ^	80 ± 5.5**	43 ± 3.7*^Δ^	31 ± 4.6^Δ^

Results are expressed as mean ± SE

*TC* total cholesterol, *TG* triglyceride, *LDL-C* low-density lipoprotein cholesterol, *HDL-C* high density lipoprotein cholesterol, *Ins* insulin, *DT* *db/db*/TNF-α^−/−^, *WT* wild-type mice

* Different at *P* < 0.05 to WT mice, ** different at *P* < 0.01 to WT mice, ^Δ^ different at *P* < 0.05 to *db/db* mice (*n* = 9/group, at each age)

### Expression of key genes involved in Wnt/β-catenin signaling and adipogenesis

**Fig. 3:**
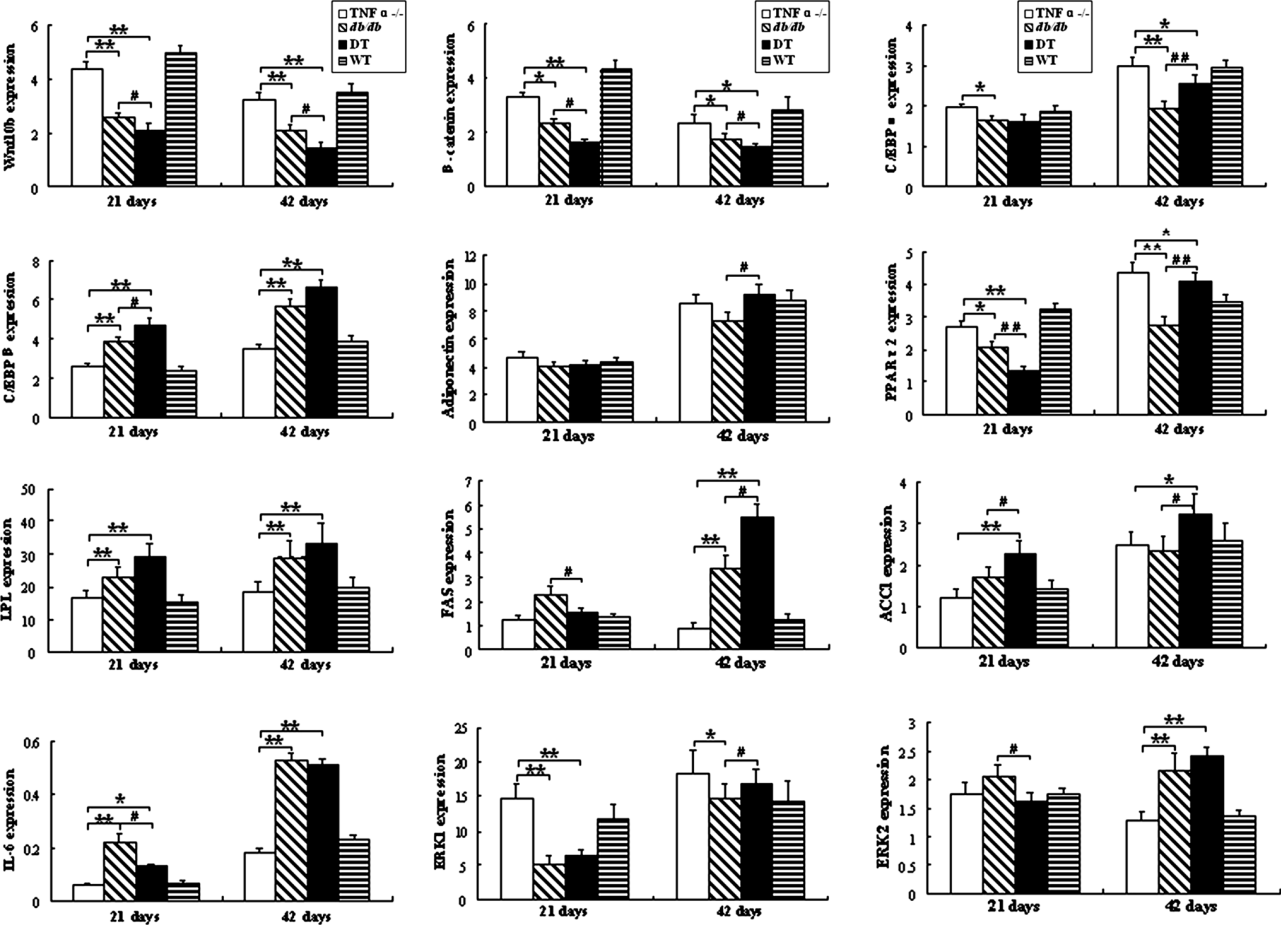
mRNA levels of Wnt/β-catenin signaling pathway and adipogenic genes in inguinal fat detected by qPCR. mRNA levels of the target gene are normalized to β-actin. *, **P < 0.05 and *P* < 0.01 significance relative to TNFα^−/−^ mice, respectively. ^#^ *P* < 0.05 significance relative to *db/db* mice. Values are expressed as the form of mean ± SEM

As shown in Fig. 3, expression of Wnt10b, β-catenin, PPARγ2, and ERK1 in eWAT are persistently decreasing since 21-day-old for obese (DT and *db/db*) mice compared with lean (TNFα^−/−^ and WT) mice (*P* < 0.05), and the lowest group is the DT one (*P* < 0.05). In addition, compared with *db/db* mice, the levels of FAS, IL-6, and ERK2 of DT mice are all significantly lower at 21-day-old (*P* < 0.05). On the other hand, the expression of C/EBPβ, adiponectin, LPL, FAS, ACCI, ERK2, and IL-6 displays a significantly high level for obese mice, and even higher for the DT group, especially at older age (*P* < 0.05, or *P* < 0.01, respectively).

### Level of proteins involved in Wnt/β-catenin signaling and adiogenesis

**Fig. 4:**
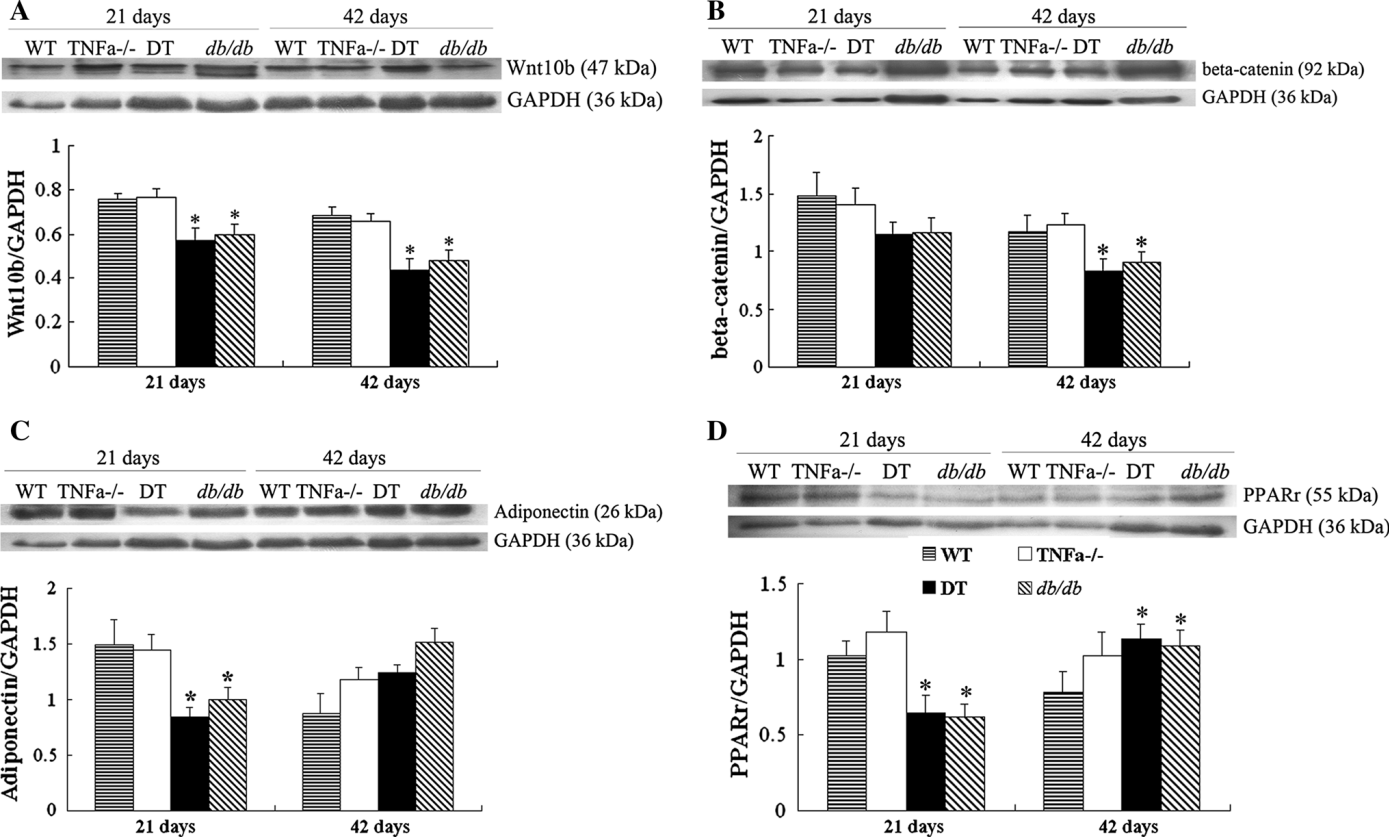
Protein levels of Wnt10b (**a**), β-catenin (**b**), adiponectin (**c**), and PPARγ2 (**d**) in the inguinal fat of the four groups of mice at each age point. Histogram from densitometric analysis is expressed as arbitrary units in the form of mean ± SEM, and normalized to the GAPDH control, respectively. *, **P < 0.05 and *P* < 0.01 significance for DT mice relative to *db/db* mice or TNFα^−/−^ mice. For per group, *N* = 12

The levels of Wnt10b and β-catenin are lower for obese mice compared with lean mice at all ages, with the lowest for the DT group (Fig. 4a, b, *P* < 0.05). Adiponectin and PPARγ2 also begin to decrease since 21-day-old for obese mice, and then increase at 42-day-old (Fig. 4c, d, *P* < 0.05). Moreover, IL-6 was down-regulated at 21-day-old for DT mice versus *db/db* mice (data not shown).

## Discussion

Our results clearly demonstrated the obese DT mice developed more severe obesity than the gender- and age-matched obese *db/db* mice, due to white adipocytes hypertrophy. However, TNFα^−/−^ and WT mice had no tendency of obesity on a chow diet, even at 6-month-old (data not shown). These findings extended our knowledge that loss of TNFα function could accelerate obesity in genetic obese background from adolescent mice on a chow diet.

It was reported that adipocytes from the expression of obese animals (*ob/ob*, *db/db* mice, and fa/fa Zucker rats) markedly increased the amount of TNFα [3]. Several studies demonstrated that the chronic and graduated elevation of TNFα in obese subjects promoted modifications in secretory function of adipocyte such as inhibiting fat synthesis through promotion of lipolysis, which caused resistance to the antilipolytic effects of insulin in adipocytes and inhibited preadipocytes proliferation and adipogenic differentiation [2, 3, 6, 8, 9, 14, 20]. However, differentiation of primary preadipocytes, either from obese mice or lean mice, was significantly inhibited after TNFα treatment (our unpublished data), which indicated that TNFα might play a role of negative feedback molecule inhibiting adipogenesis. Unfortunately, contributions of TNFα to obese development still remained unclear as mentioned earlier. According to previous work, the body weight and epididymal fat of TNFα mutant and WT mice were similar when feeding with a chow diet while both of the mice developed obesity when feeding with a high fat diet for several months [6], which was consistent with our study. Moreover, Uysal KT et al. showed that no significant difference existed in body weight and body composition between the obese TNFα deficient mice and obese *ob/ob* mice [6]. In present work, however, we found that body weight and eWAT mass in DT mice were significantly increased compared with those of *db/db* mice. This might be attributed to the intact leptin (in our work) or leptin receptor (in their work), which could still remained functions through related pathways. Interestingly, these results suggested that since metabolic situation of genetic obese mice or diet induced obese mice was different from that of lean subjects, the absence of TNFα did not significantly affect the body weight gain of mice when feeding with a chow diet, but affected the obese mice on a chow diet or lean subjects on a high fat diet.

For human beings, the obese state is characterized by the increase in the serum levels of multiple cytokines, such as TNFα, IL-1, IL-6, IL-8, soluble TNF receptor 1, and macrophage inflammatory protein-1α, all of which decline with weight loss [3, 21, 22]. Adipocytes from obese individuals exhibited that higher basal lipolysis might be caused by elevated levels of inflammatory cytokines, especially TNFα [23–27]. In the present study, obese DT mice were found to be fattier than other groups, which indicated that the loss of TNFα function proliferation and maturation of eWAT in the DT mice played a dominate role compared with lipolytic effects.

To determine the effects of TNFα deficiency on the signaling capacity of the canonical Wnt pathway in vivo, we examined the expression levels of Wnt10b, β-cantenin, and adipogenic molecules in the mice. Not surprisingly, we have found that obese *db/db* mice lacking TNFα function developed early onset and severe obesity compared with *db/db* mice; compared with TNFα^−/−^ mice, *db/db* mice showed that key molecules in Wnt/β-catenin signaling were down-regulated both in mRNA and protein levels from adolescent age, while adipogenic markers were up-regulated. In addition, compared with *db/db* mice, DT mice presented high mRNA levels of C/EBPα, PPARγ2, LPL, FAS, ACCI, and adiponectin, but low in Wnt/β-catenin signaling. Wnt/β-catenin signaling inhibited the differentiation of preadipocytes through activating Wnt10b produced by preadipocytes [24, 25], which prevented its own differentiation [17] and could be elevated by TNFα stimulation [14]. In the present study, Wnt10b and β-catenin were decreased both in DT mice and TNFα^−/−^ mice and much lower in DT mice than in TNFα^−/−^ mice, which suggested Wnt/β-catenin signaling was inhibited due to TNFα deficiency either in the obese or lean mice. Our results indicated that TNFα–Wnt/β-catenin–adipogenic pathway played important roles in adipocyte differentiation, maturation and obese development, and that TNFα deficiency inhibited Wnt/β-catenin signaling and up-regulation adipogenic molecules.

Adipocytes differentiation related transcription factors, such as PPARγ, play a crucial role in the induction of adipocyte differentiation and lipid accumulating cells. In our study, PPARγ2 in DT mice was higher than that in the *db/db* mice at 42-day-old, which indicated that TNFα deficiency had an influence on PPARγ expression. Besides, activation of Wnt signaling also prevented the normal activation of C/EBPα, but not that of C/EBPβ and C/EBPδ, as reported in the study for both TNFα and IL-6 [28]. Obesity-related increase in IL-6 may also contribute to the obesity, and it has been described that increased IL-6 production was linked with high TNFα expression [29–31]. In the present study, IL-6 levels in mRNA and protein both showed significantly up-regulated tendency in obese mice compared with lean mice, but were significantly low in DT mice compared with *db/db* mice at 21-day-old. The results indicated that obesity promoted IL-6 production, but did not amend spontaneously due to TNFα deficiency.

There are several mechanisms that may lead to acceleration of adipocytes proliferation, and maturation, and lipid accumulation in adipocyte, which result in body weight gain of DT mice compared with *db/db* mice. One possible reason may be due to the decreased lipolysis, as well as apoptosis with increased lipid accumulation of adipocytes in TNFα deficient obese mice. Another possibility is that TNFα deficiency reduces the inflammatory response in the systemic [4] and local eWAT, and prevents the infiltration of macrophages into eWAT to produce inflammatory cytokines. To our knowledge, it is the first report that deficiency of TNFα on an obese background can promote obesity development in young genetic obese mice, and our results show that TNFα negatively regulates adipogenesis in obese subjects by, at least in part, inhibiting Wnt/β-catenin signaling.

In summary, our evidences of obesity promotion in TNFα deficient obese mice proposed that TNFα might correlate with other signaling pathways in obese development, although the relative role of TNFα in adipogenic network such as chemerin/CMKLR signaling still remained evaluated. To address this possibility, further in vivo and in vitro study should be done.

## Electronic supplementary material

Below is the link to the electronic supplementary material.

Supplementary Table 1 Primers for qPCR analysis (DOC 30 kb)
