# Supplementary material for: Loss of the TNFα function inhibits Wnt/β-catenin signaling, exacerbates obesity development in adolescent spontaneous obese mice
Source: Mol Cell Biochem. 2014 Feb 13;391(1):59–66. doi: 10.1007/s11010-014-1987-5 (PMC4006126; doi:10.1007/s11010-014-1987-5)
Supplement: Supplementary file 1 — Supplementary Table 1 Primers for qPCR analysis (DOC 30 kb) [file 11010_2014_1987_MOESM1_ESM.doc]

**Supplementary Table**  Primers for qPCR analysis

| **Gene** | **GenBank No.** | **Forward primer (5’-3’)** | **Reverse primer (5’-3’)** |
| --- | --- | --- | --- |
| Wnt10b | NM011718 | cgaaggataatagcaggcat | gtcacccgaggtcccata |
| β-catenin | NM007614 | accctgaggaagaagatgttgac | ttgcgtgaaggactgggaa |
| C/EBPα | NM007678 | caggaggaagatacaggaagc | tctccatgaactcacccagg |
| C/EBPβ | NM009883 | acacgggactgacgcaacac | aaccccgcaggaacatcttt |
| adiponectin | NM009605 | ccaatgtacccattcgcttta | gaggctcaccttcacatctttc |
| PPARγ 2 | NM011146 | gtcatcctgctcttctttctcg | atggcgtcccttctcctgt |
| LPL | NM008509 | cttcttgatttacacggaggt | atggcatttcacaaacactg |
| FAS | NM007987 | acccaagcggtctggaaag | cggatgcctctgaaccactc |
| ACC1 | NM133360 | tgcctatgaactcaacagcg | tggggagtcacagaagcag |
| IL-6 | NM031168 | accacggccttccctacttc | ctcatttccacgatttcccag |
| ERK1 | NM133360 | gagaatgttataggcatccgaga | gtcgcaggtggtgttgataag |
| ERK2 | NM008509 | tcccaaatgctgactccaaa | agagcctgttcaacttcaatcct |
| β-actin | NM007393 | cctctatgccaacacagtgc | gtactcctgcttgctgatcc |

# Maolei Gong et al
